# Supplementary material for: Angiogenic Activity of Cytochalasin B-Induced Membrane Vesicles of Human Mesenchymal Stem Cells
Source: Cells. 2019 Dec 30;9(1):95. doi: 10.3390/cells9010095 (PMC7016674; doi:10.3390/cells9010095)
Supplement: Supplementary file 1 [file cells-09-00095-s001.pdf]

Angiogenic activity of cytochalasin B-induced membrane vesicles of human mesenchymal stem cells

M.O. Gomzikova[1,2], M.N. Zhuravleva[1], V.V. Vorobev[1], I.I. Salafutdinov[1], A.V.Laikov[1], S.K. Kletukhina[1], E.V. Martynova[1], L.G. Tazetdinova[1], A.I.Ntekim[3,4], S.F. Khaiboullina[1,5], A.A. Rizvanov[1,2]

[1]Kazan Federal University, Kazan, 420008, Russia

[2]M.M. Shemyakin–Yu.A. Ovchinnikov Institute of Bioorganic Chemistry of the Russian Academy of Sciences, Moscow, 117997, Russia

[3]Faculty of Medicine and Health Sciences, University of Nottingham, LE12 5RD, UK

[4]Department of Radiation Oncology, College of Medicine, University of Ibadan Nigeria

[5]Department of Microbiology and Immunology, University of Nevada, Reno School of Medicine, Reno, NV, USA

Supplementary Data

RESULTS

SI Table 1. Proteome analysis of CIMVs-MSCs

	Protein	Subcellular localization
1.	14-3-3 protein gamma<br>OS=Homo sapiens GN=YWHAG PE=1 SV=2	Cytoplasm
2.	14-3-3 protein zeta/delta<br>OS=Homo sapiens GN=YWHAZ PE=1 SV=1	Cytoplasm, melanosome
3.	3-hydroxyacyl-CoA dehydrogenase type-2<br>OS=Homo sapiens GN=HSD17B10 PE=1 SV=3	Mitochondrion
4.	3-ketoacyl-CoA thiolase, mitochondrial<br>OS=Homo sapiens GN=ACAA2 PE=1 SV=2	Mitochondrion
5.	40S ribosomal protein S18<br>OS=Homo sapiens GN=RPS18 PE=1 SV=3	Cytoplasm
6.	40S ribosomal protein SA<br>OS=Homo sapiens GN=RPSA PE=1 SV=4	Cell membrane, cytoplasm, nucleus
7.	60S ribosomal protein L15<br>OS=Homo sapiens GN=RPL15 PE=1 SV=2	Cytoplasm
8.	60S ribosomal protein L18<br>OS=Homo sapiens GN=RPL18 PE=1 SV=2	Cytoplasm

9.	Acetyl-CoA acetyltransferase, mitochondrial<br>OS=Homo sapiens GN=ACAT1 PE=1 SV=1	Mitochondrion
10.	Actin-related protein 2<br>OS=Homo sapiens GN=ACTR2 PE=1 SV=1	Cytoplasm, cytoskeleton, cell projection
11.	Actin-related protein 2/3 complex subunit 1B<br>OS=Homo sapiens GN=ARPC1B PE=1 SV=3	Cytoplasm, cytoskeleton
12.	Actin-related protein 2/3 complex subunit 4<br>OS=Homo sapiens GN=ARPC4 PE=1 SV=3	Cytoplasm, cytoskeleton, cell projection
13.	Actin-related protein 2/3 complex subunit 5<br>OS=Homo sapiens GN=ARPC5 PE=1 SV=3	Cytoplasm, cytoskeleton, cell projection
14.	Actin-related protein 2/3 complex subunit 5-like protein<br>OS=Homo sapiens GN=ARPC5L PE=1 SV=1	Cytoplasm, cytoskeleton
15.	Actin-related protein 3<br>OS=Homo sapiens GN=ACTR3 PE=1 SV=3	Cytoplasm, cytoskeleton, cell projection
16.	Actin-related protein 3B<br>OS=Homo sapiens GN=ACTR3B PE=1 SV=1	Cytoplasm, cytoskeleton, cell projection
17.	Adenylate kinase isoenzyme 1<br>OS=Homo sapiens GN=AK1 PE=1 SV=3	Cytoplasm
18.	Alpha-centractin<br>OS=Homo sapiens GN=ACTR1A PE=1 SV=1	Cytoplasm, cytoskeleton, microtubule organizing center, centrosome
19.	AP-2 complex subunit beta<br>OS=Homo sapiens GN=AP2B1 PE=1 SV=1	Cell membrane, coated pit Peripheral membrane protein, cytoplasmic side
20.	ATP synthase subunit O, mitochondrial<br>OS=Homo sapiens GN=ATP5O PE=1 SV=1	Mitochondrion, mitochondrion inner membrane
21.	ATP-citrate synthase<br>OS=Homo sapiens GN=ACLY PE=1 SV=3	Cytoplasm
22.	Beta-galactosidase<br>OS=Homo sapiens GN=GLB1 PE=1 SV=2	Lysosome, cytoplasm › perinuclear region
23.	Bifunctional glutamate/proline--tRNA ligase<br>OS=Homo sapiens GN=EPRS PE=1 SV=5	Cytoplasm
24.	Caldesmon<br>OS=Homo sapiens GN=CALD1 PE=1 SV=3	Cytoplasm, cytoskeleton, cytoplasm myofibril
25.	cAMP-dependent protein kinase type I-alpha regulatory subunit<br>OS=Homo sapiens GN=PRKAR1A PE=1 SV=1	Cell membrane
26.	Carbonyl reductase [NADPH] 1<br>OS=Homo sapiens GN=CBR1 PE=1 SV=3	Cytoplasm
27.	Carbonyl reductase [NADPH] 3<br>OS=Homo sapiens GN=CBR3 PE=1 SV=3	Cytoplasm
28.	Catenin alpha-2<br>OS=Homo sapiens GN=CTNNA2 PE=1 SV=5	Cell membrane, peripheral membrane protein, cytoplasmic side, cytoplasm, cytoplasm, cytoskeleton, cell junction, adherens junction, cell projection, axon, nucleus
29.	CD9 antigen<br>OS=Homo sapiens GN=CD9 PE=1 SV=4	Membrane, multi-pass membrane protein, cell membrane, multi-pass membrane protein

30.	Cellular retinoic acid-binding protein 2 OS=Homo sapiens GN=CRABP2 PE=1 SV=2	Cytoplasm, endoplasmic reticulum, nucleus
31.	Chloride intracellular channel protein 1 OS=Homo sapiens GN=CLIC1 PE=1 SV=4	Nucleus, nucleus membrane, single-pass membrane protein, cytoplasm, cell membrane, single-pass membrane protein
32.	Chloride intracellular channel protein 4 OS=Homo sapiens GN=CLIC4 PE=1 SV=4	Cytoplasm, cytoskeleton, microtubule organizing center, centrosome, cytoplasmic vesicle membrane, single-pass membrane protein, nucleus matrix, cell membrane, single-pass membrane protein, mitochondrion, cell junction
33.	Cofilin-1 OS=Homo sapiens GN=CFL1 PE=1 SV=3	Nucleus matrix, cytoplasm, cytoskeleton, cell projection, ruffle membrane, peripheral membrane protein, cytoplasmic side, cell projection, lamellipodium membrane, peripheral membrane protein, cytoplasmic side
34.	Cofilin-2 OS=Homo sapiens GN=CFL2 PE=1 SV=1	Nucleus matrix, cytoplasm, cytoskeleton
35.	Copine-3 OS=Homo sapiens GN=CPNE3 PE=1 SV=1	Nucleus, cytoplasm, cell membrane, cell junction, cell junction, focal adhesion
36.	Coronin-1B OS=Homo sapiens GN=CORO1B PE=1 SV=1	Cytoplasm, cytoskeleton
37.	Creatine kinase B-type OS=Homo sapiens GN=CKB PE=1 SV=1	Cytoplasm
38.	Cullin-associated NEDD8-dissociated protein 1 OS=Homo sapiens GN=CAND1 PE=1 SV=2	Cytoplasm, nucleus
39.	Cytochrome c oxidase subunit 2 OS=Homo sapiens GN=MT-CO2 PE=1 SV=1	Mitochondrion inner membrane, multi-pass membrane protein
40.	Cytoplasmic dynein 1 heavy chain 1 OS=Homo sapiens GN=DYNC1H1 PE=1 SV=5	Cytoplasm, cytoskeleton
41.	Cytosol aminopeptidase OS=Homo sapiens GN=LAP3 PE=1 SV=3	Cytoplasm
42.	Dihydropyrimidinase-related protein 1 OS=Homo sapiens GN=CRMP1 PE=1 SV=1	Cytoplasm, cytoplasm, cytoskeleton, microtubule organizing center, centrosome, cytoplasm, cytoskeleton, spindle
43.	Dihydropyrimidinase-related protein 3 OS=Homo sapiens GN=DPYSL3 PE=1 SV=1	Cytoplasm, cell projection, growth cone
44.	Early endosome antigen 1 OS=Homo sapiens GN=EEA1 PE=1 SV=2	Cytoplasm, early endosome membrane, peripheral membrane protein
45.	Eukaryotic translation initiation factor 3 subunit F	Cytoplasm

	OS=Homo sapiens GN=EIF3F PE=1 SV=1	
46.	Fatty acid-binding protein, heart<br>OS=Homo sapiens GN=FABP3 PE=1 SV=4	Cytoplasm
47.	Flavin reductase (NADPH)<br>OS=Homo sapiens GN=BLVRB PE=1 SV=3	Cytoplasm
48.	Gamma-adducin<br>OS=Homo sapiens GN=ADD3 PE=1 SV=1	Cytoplasm, cytoskeleton, cell membrane, peripheral membrane protein, cytoplasmic side
49.	Glutathione S-transferase kappa 1<br>OS=Homo sapiens GN=GSTK1 PE=1 SV=3	Peroxisome
50.	Glycine--tRNA ligase<br>OS=Homo sapiens GN=GARS PE=1 SV=3	Cytoplasm, mitochondrion, cell projection, axon
51.	Golgi-associated plant pathogenesis-related protein 1<br>OS=Homo sapiens GN=GLIPR2 PE=1 SV=3	Golgi apparatus membrane, lipid-anchor
52.	Guanine nucleotide-binding protein G(i) subunit alpha-1<br>OS=Homo sapiens GN=GNAI1 PE=1 SV=2	Nucleus, cytoplasm, cell membrane, peripheral membrane protein, cytoplasmic side, cytoplasm, cytoskeleton, microtubule organizing center, centrosome, membrane, lipid-anchor
53.	Guanine nucleotide-binding protein subunit alpha-11<br>OS=Homo sapiens GN=GNA11 PE=1 SV=2	Cell membrane, lipid-anchor, cytoplasm
54.	Guanine nucleotide-binding protein subunit alpha-13<br>OS=Homo sapiens GN=GNA13 PE=1 SV=2	Membrane, lipid-anchor, melanosome, cytoplasm, nucleus
55.	Heat shock 70 kDa protein 4<br>OS=Homo sapiens GN=HSPA4 PE=1 SV=4	Cytoplasm
56.	Heme-binding protein 1<br>OS=Homo sapiens GN=HEBP1 PE=1 SV=1	Cytoplasm
57.	HLA class I histocompatibility antigen, A-23 alpha chain<br>OS=Homo sapiens GN=HLA-A PE=1 SV=1	Membrane, single-pass type I membrane protein
58.	HLA class I histocompatibility antigen, A-32 alpha chain<br>OS=Homo sapiens GN=HLA-A PE=2 SV=2	Membrane, single-pass type I membrane protein
59.	HLA class I histocompatibility antigen, A-68 alpha chain<br>OS=Homo sapiens GN=HLA-A PE=1 SV=4	Membrane; single-pass type I membrane protein
60.	HLA class I histocompatibility antigen, A-80 alpha chain<br>OS=Homo sapiens GN=HLA-A PE=2 SV=1	Membrane, single-pass type I membrane protein
61.	HLA class I histocompatibility antigen, B-27 alpha chain<br>OS=Homo sapiens GN=HLA-B PE=1 SV=2	Membrane, single-pass type I membrane protein
62.	HLA class I histocompatibility antigen, Cw-12 alpha chain<br>OS=Homo sapiens GN=HLA-C PE=1 SV=2	Membrane, single-pass type I membrane protein
63.	Importin-5<br>OS=Homo sapiens GN=IPO5 PE=1 SV=4	Cytoplasm, nucleus, nucleus, nucleolus
64.	Insulin-like growth factor 2 mRNA-binding protein 2<br>OS=Homo sapiens GN=IGF2BP2 PE=1 SV=2	Nucleus, cytoplasm

65.	Intercellular adhesion molecule 1<br>OS=Homo sapiens GN=ICAM1 PE=1 SV=2	Membrane, single-pass type I membrane protein
66.	Interferon-induced GTP-binding protein Mx1<br>OS=Homo sapiens GN=MX1 PE=1 SV=4	Cytoplasm, endoplasmic reticulum membrane, peripheral membrane protein, cytoplasmic side, cytoplasm, perinuclear region
67.	L-lactate dehydrogenase B chain<br>OS=Homo sapiens GN=LDHB PE=1 SV=2	Cytoplasm
68.	Lon protease homolog, mitochondrial<br>OS=Homo sapiens GN=LONP1 PE=1 SV=2	Mitochondrion matrix
69.	Microsomal glutathione S-transferase 3<br>OS=Homo sapiens GN=MGST3 PE=1 SV=1	Endoplasmic reticulum membrane, multi-pass membrane protein, microsome membrane, multi-pass membrane protein, microsome membrane, lipid-anchor
70.	Microtubule-associated protein 1B<br>OS=Homo sapiens GN=MAP1B PE=1 SV=2	Cytoplasm, cytoskeleton, cytoplasm, cell junction, synapse, cell projection, dendritic spine
71.	Mitogen-activated protein kinase 1<br>OS=Homo sapiens GN=MAPK1 PE=1 SV=3	Cytoplasm, cytoskeleton, spindle, nucleus, cytoplasm, cytoskeleton, microtubule organizing center, centrosome, cytoplasm
72.	Myelin protein zero-like protein 1<br>OS=Homo sapiens GN=MPZL1 PE=1 SV=1	Membrane, single-pass type I membrane protein
73.	Myeloid-associated differentiation marker<br>OS=Homo sapiens GN=MYADM PE=1 SV=2	Membrane, multi-pass membrane protein
74.	Nicotinamide phosphoribosyltransferase<br>OS=Homo sapiens GN=NAMPT PE=1 SV=1	Nucleus, cytoplasm, secreted
75.	Peroxiredoxin-5, mitochondrial<br>OS=Homo sapiens GN=PRDX5 PE=1 SV=4	Isoform mitochondrial, mitochondrion, isoform cytoplasmic+peroxisomal, cytoplasm, peroxisome
76.	Phosphatidylethanolamine-binding protein 1<br>OS=Homo sapiens GN=PEBP1 PE=1 SV=3	Cytoplasm
77.	Plasma membrane calcium-transporting ATPase 1<br>OS=Homo sapiens GN=ATP2B1 PE=1 SV=3	Cell membrane, multi-pass membrane protein
78.	Plasma membrane calcium-transporting ATPase 4<br>OS=Homo sapiens GN=ATP2B4 PE=1 SV=2	Cell membrane, multi-pass membrane protein, cell projection, cilium, flagellum membrane, multi-pass membrane protein
79.	Platelet-derived growth factor receptor beta<br>OS=Homo sapiens GN=PDGFRB PE=1 SV=1	Cell membrane, single-pass type I membrane protein, cytoplasmic vesicle, lysosome lumen
80.	Programmed cell death protein 6<br>OS=Homo sapiens GN=PDCD6 PE=1 SV=1	Nucleus membrane, peripheral membrane protein, endoplasmic reticulum membrane, peripheral membrane protein, nucleus, endosome

81.	Proteasome subunit beta type-1<br>OS=Homo sapiens GN=PSMB1 PE=1 SV=2	Cytoplasm, nucleus
82.	Protein disulfide-isomerase A4<br>OS=Homo sapiens GN=PDIA4 PE=1 SV=2	Endoplasmic reticulum lumen, melanosome
83.	Protein Hook homolog 3<br>OS=Homo sapiens GN=HOOK3 PE=1 SV=2	Cytoplasm, cytoskeleton, Golgi apparatus
84.	Puromycin-sensitive aminopeptidase<br>OS=Homo sapiens GN=NPEPPS PE=1 SV=2	Cytoplasm, cytosol, nucleus
85.	Putative HLA class I histocompatibility antigen, alpha chain H<br>OS=Homo sapiens GN=HLA-H PE=5 SV=3	Cell membrane, single-pass membrane protein
86.	Radixin<br>OS=Homo sapiens GN=RDX PE=1 SV=1	Cell membrane, peripheral membrane protein, cytoplasmic side, cytoplasm, cytoskeleton, cleavage furrow
87.	Ras-related protein Rab-15<br>OS=Homo sapiens GN=RAB15 PE=1 SV=1	Cell membrane, lipid-anchor, cytoplasmic side
88.	Ras-related protein Rab-5B<br>OS=Homo sapiens GN=RAB5B PE=1 SV=1	Cell membrane, lipid-anchor, cytoplasmic side, early endosome membrane, lipid-anchor, melanosome
89.	Ras-related protein R-Ras<br>OS=Homo sapiens GN=RRAS PE=1 SV=1	Cell membrane, lipid-anchor, cytoplasmic side
90.	Reticulocalbin-3<br>OS=Homo sapiens GN=RCN3 PE=1 SV=1	Endoplasmic reticulum lumen
91.	Reticulon-4<br>OS=Homo sapiens GN=RTN4 PE=1 SV=2	Endoplasmic reticulum membrane, multi-pass membrane protein
92.	Rho-associated protein kinase 2<br>OS=Homo sapiens GN=ROCK2 PE=1 SV=4	Cytoplasm, cell membrane, peripheral membrane protein, nucleus, cytoplasm, cytoskeleton, microtubule organizing center, centrosome
93.	Ribonuclease inhibitor<br>OS=Homo sapiens GN=RNH1 PE=1 SV=2	Cytoplasm
94.	Septin-6<br>OS=Homo sapiens GN=SEPT6 PE=1 SV=4	Cytoplasm, cytoplasm, cytoskeleton, spindle chromosome, centromere, kinetochore, cleavage furrow, midbody
95.	Septin-7<br>OS=Homo sapiens GN=SEPT7 PE=1 SV=2	Cytoplasm, chromosome, centromere, kinetochore, cytoplasm, cytoskeleton, spindle, cleavage furrow, midbody, cytoplasm, cytoskeleton, cilium axoneme
96.	Signal transducer and activator of transcription 1-alpha/beta<br>OS=Homo sapiens GN=STAT1 PE=1 SV=2	Cytoplasm, nucleus
97.	Sodium/potassium-transporting ATPase subunit alpha-2<br>OS=Homo sapiens GN=ATP1A2 PE=1 SV=1	Membrane, multi-pass membrane protein, cell membrane, multi-pass membrane protein
98.	Sodium/potassium-transporting ATPase subunit alpha-4<br>OS=Homo sapiens GN=ATP1A4 PE=1 SV=3	Cell membrane, multi-pass membrane protein

99.	Spectrin beta chain, erythrocyte<br>OS=Homo sapiens GN=SPTB PE=1 SV=5	Cytoplasm, cytoskeleton, cytoplasm, cell cortex
100.	Threonine--tRNA ligase, cytoplasmic<br>OS=Homo sapiens GN=TARS PE=1 SV=3	Cytoplasm
101.	Titin<br>OS=Homo sapiens GN=TTN PE=1 SV=4	Cytoplasm, nucleus
102.	Tripeptidyl-peptidase 1<br>OS=Homo sapiens GN=TPP1 PE=1 SV=2	Lysosome, melanosome
103.	Tropomodulin-3<br>OS=Homo sapiens GN=TMOD3 PE=1 SV=1	Cytoplasm, cytoskeleton
104.	Tubulin alpha-3E chain<br>OS=Homo sapiens GN=TUBA3E PE=1 SV=2	Cytoplasm, cytoskeleton
105.	Tubulin alpha-8 chain<br>OS=Homo sapiens GN=TUBA8 PE=1 SV=1	Cytoplasm, cytoskeleton
106.	Tubulin beta-2B chain<br>OS=Homo sapiens GN=TUBB2B PE=1 SV=1	Cytoplasm, cytoskeleton
107.	Tubulin beta-8 chain<br>OS=Homo sapiens GN=TUBB8 PE=1 SV=2	Cytoplasm, cytoskeleton
108.	Unconventional myosin-Ic<br>OS=Homo sapiens GN=MYO1C PE=1 SV=4	Isoform 1: cytoplasm, nucleus;<br>Isoform 2: cytoplasm, cell membrane, peripheral membrane protein, cytoplasmic side, cell projection, stereocilium membrane, cell projection, ruffle, cytoplasmic vesicle;<br>Isoform 3: nucleus, nucleoplasm, nucleus, nucleolus, nucleus, nuclear pore complex
109.	Utrophin<br>OS=Homo sapiens GN=UTRN PE=1 SV=2	Cell junction, synapse, postsynaptic cell membrane, peripheral membrane protein, cytoplasmic side, cytoplasm, cytoskeleton
110.	WD repeat-containing protein 1<br>OS=Homo sapiens GN=WDR1 PE=1 SV=4	Cytoplasm, cytoskeleton, cell projection, podosome

SI Table 2. Proteome analysis of MSCs

	Protein	Subcellular localization
1.	116 kDa U5 small nuclear ribonucleoprotein component OS=Homo sapiens GN=EFTUD2 PE=1 SV=1	Intracellular ribonucleoprotein complex, viral nucleocapsid
2.	26S proteasome non-ATPase regulatory subunit 1 OS=Homo sapiens GN=PSMD1 PE=1 SV=2	Cytosol, extracellular exosome, membrane, nucleoplasm, nucleus, proteasome accessory complex, proteasome complex, proteasome regulatory particle, proteasome regulatory particle, base subcomplex, proteasome storage granule, cytosol, extracellular exosome, membrane, nucleoplasm, nucleus, proteasome accessory complex, proteasome complex, proteasome regulatory particle, proteasome regulatory particle, base subcomplex, proteasome storage granule
3.	26S proteasome non-ATPase regulatory subunit 11 OS=Homo sapiens GN=PSMD11 PE=1 SV=3	Nucleus, cytoplasm, cytosol
4.	26S proteasome non-ATPase regulatory subunit 7 OS=Homo sapiens GN=PSMD7 PE=1 SV=2	Cytosol, extracellular exosome, membrane, nucleoplasm, nucleus, proteasome complex, proteasome regulatory particle
5.	2-oxoglutarate dehydrogenase-like, mitochondrial OS=Homo sapiens GN=OGDHL PE=1 SV=3	Mitochondrion matrix
6.	60S ribosomal protein L12 OS=Homo sapiens GN=RPL12 PE=1 SV=1	Cytosol, cytosolic large ribosomal subunit, extracellular exosome, focal adhesion, membrane
7.	60S ribosomal protein L4 OS=Homo sapiens GN=RPL4 PE=1 SV=5	Cytoplasm, cytosol, cytosolic large ribosomal subunit, extracellular exosome, focal adhesion, intracellular ribonucleoprotein complex, membrane, nucleolus, nucleus
8.	60S ribosomal protein L5 OS=Homo sapiens GN=RPL5 PE=1 SV=3	Cytoplasm, nucleus, nucleolus
9.	6-phosphogluconate dehydrogenase, decarboxylating OS=Homo sapiens GN=PGD PE=1 SV=3	Cytoplasm
10.	Adenosylhomocysteinase OS=Homo sapiens GN=AHCY PE=1 SV=4	Endoplasmic reticulum, cytoplasm, cytosol, microsome, apical cell membrane
11.	Adipocyte plasma membrane-associated protein OS=Homo sapiens GN=APMAP PE=1 SV=2	Membrane
12.	ADP/ATP translocase 3 OS=Homo sapiens GN=SLC25A6 PE=1 SV=4	Mitochondrion inner membrane, multi-pass membrane protein
13.	ADP-ribosylation factor-like protein 8B OS=Homo sapiens GN=ARL8B PE=1 SV=1	Late endosome membrane, lysosome membrane, cytoplasm, cytoskeleton, spindle
14.	Aflatoxin B1 aldehyde reductase member 2 OS=Homo sapiens GN=AKR7A2 PE=1 SV=3	Golgi apparatus, cytoplasm

15.	Aldo-keto reductase family 1 member C2<br>OS=Homo sapiens GN=AKR1C2 PE=1 SV=3	Cytoplasm
16.	Alpha-soluble NSF attachment protein<br>OS=Homo sapiens GN=NAPA PE=1 SV=3	Membrane, peripheral membrane protein
17.	Atlastin-3<br>OS=Homo sapiens GN=ATL3 PE=1 SV=1	Endoplasmic reticulum membrane, multi-pass membrane protein
18.	ATP-dependent RNA helicase A<br>OS=Homo sapiens GN=DHX9 PE=1 SV=4	Nucleus speckle, cytoplasm, mitochondrion outer membrane
19.	Coatomer subunit beta<br>OS=Homo sapiens GN=COPB1 PE=1 SV=3	Cytoplasm, Golgi apparatus membrane, peripheral membrane protein, cytoplasmic side, cytoplasmic vesicle, COPI-coated vesicle membrane, peripheral membrane protein, cytoplasmic side, cell membrane, endoplasmic reticulum-Golgi intermediate compartment
20.	Coatomer subunit delta<br>OS=Homo sapiens GN=ARCN1 PE=1 SV=1	Cytoplasm, Golgi apparatus membrane, peripheral membrane protein, cytoplasmic side, cytoplasmic vesicle, COPI-coated vesicle membrane, peripheral membrane protein, cytoplasmic side, cell membrane, endoplasmic reticulum-Golgi intermediate compartment
21.	Coatomer subunit epsilon<br>OS=Homo sapiens GN=COPE PE=1 SV=3	Cytoplasm, Golgi apparatus membrane, peripheral membrane protein, cytoplasmic side, cytoplasmic vesicle, COPI-coated vesicle membrane, peripheral membrane protein, cytoplasmic side, cell membrane, endoplasmic reticulum-Golgi intermediate compartment
22.	Collagen alpha-1(I) chain<br>OS=Homo sapiens GN=COL1A1 PE=1 SV=5	Secreted, extracellular space, extracellular matrix
23.	Collagen alpha-2(I) chain<br>OS=Homo sapiens GN=COL1A2 PE=1 SV=7	Secreted, extracellular space, extracellular matrix
24.	Core histone macro-H2A.1<br>OS=Homo sapiens GN=H2AFY PE=1 SV=4	Nucleus, chromosome
25.	Cytochrome b5<br>OS=Homo sapiens GN=CYB5A PE=1 SV=2	Isoform 1: endoplasmic reticulum membrane, lipid-anchor, cytoplasmic side, mitochondrion outer membrane, lipid-anchor, cytoplasmic side<br>Isoform 2: cytoplasm
26.	DBIRD complex subunit KIAA1967<br>OS=Homo sapiens GN=KIAA1967 PE=1 SV=2	Nucleus, cytoplasm
27.	Delta-1-pyrroline-5-carboxylate synthase<br>OS=Homo sapiens GN=ALDH18A1 PE=1 SV=2	Mitochondrion inner membrane
28.	Desmin<br>OS=Homo sapiens GN=DES PE=1 SV=3	Cytoplasm, myofibril, sarcomere, Z line, cytoplasm, cell membrane, sarcolemma
29.	Diablo homolog, mitochondrial<br>OS=Homo sapiens GN=DIABLO PE=1 SV=1	Mitochondrion
30.	Dynactin subunit 1<br>OS=Homo sapiens GN=DCTN1 PE=1 SV=3	Cytoplasm, cytoplasm, cytoskeleton, cytoplasm, cytoskeleton, microtubule organizing center, centrosome

31.	Dynactin subunit 2<br>OS=Homo sapiens GN=DCTN2 PE=1 SV=4	Cytoplasm, cytoskeleton, microtubule organizing center, centrosome, membrane, peripheral membrane protein
32.	ELAV-like protein 1<br>OS=Homo sapiens GN=ELAVL1 PE=1 SV=2	Cytoplasm, nucleus
33.	Enoyl-CoA delta isomerase 1, mitochondrial<br>OS=Homo sapiens GN=ECI1 PE=1 SV=1	Mitochondrion matrix
34.	Eukaryotic translation initiation factor 2 subunit 1<br>OS=Homo sapiens GN=EIF2S1 PE=1 SV=3	Cytoplasmic granule
35.	Eukaryotic translation initiation factor 5<br>OS=Homo sapiens GN=EIF5 PE=1 SV=2	Cytoplasm, nucleus, endoplasmic reticulum membrane, peripheral membrane protein, cytoplasmic side, nucleus, nuclear pore complex
36.	Far upstream element-binding protein 2<br>OS=Homo sapiens GN=KHSRP PE=1 SV=4	Nucleus, cytoplasm
37.	Glia-derived nexin<br>OS=Homo sapiens GN=SERPINE2 PE=1 SV=1	Secreted, extracellular space
38.	Guanine nucleotide-binding protein G(q) subunit alpha<br>OS=Homo sapiens GN=GNAQ PE=1 SV=4	Cell membrane, lipid-anchor
39.	Heat shock protein 75 kDa, mitochondrial<br>OS=Homo sapiens GN=TRAP1 PE=1 SV=3	Mitochondrion, mitochondrion inner membrane, mitochondrion matrix
40.	Heterogeneous nuclear ribonucleoprotein A1-like 2<br>OS=Homo sapiens GN=HNRNPA1L2 PE=2 SV=2	Nucleus, cytoplasm
41.	Heterogeneous nuclear ribonucleoprotein C-like 1<br>OS=Homo sapiens GN=HNRNPCL1 PE=1 SV=1	Nucleus
42.	Heterogeneous nuclear ribonucleoprotein D0<br>OS=Homo sapiens GN=HNRNPD PE=1 SV=1	Nucleus, cytoplasm
43.	Heterogeneous nuclear ribonucleoprotein F<br>OS=Homo sapiens GN=HNRNPF PE=1 SV=3	Nucleus, nucleoplasm
44.	Heterogeneous nuclear ribonucleoprotein H<br>OS=Homo sapiens GN=HNRNPH1 PE=1 SV=4	Nucleus, nucleoplasm
45.	Heterogeneous nuclear ribonucleoprotein K<br>OS=Homo sapiens GN=HNRNPK PE=1 SV=1	Cytoplasm, nucleus, nucleoplasm, cell projection, podosome
46.	Heterogeneous nuclear ribonucleoprotein L<br>OS=Homo sapiens GN=HNRNPL PE=1 SV=2	Nucleus, nucleoplasm, cytoplasm
47.	Heterogeneous nuclear ribonucleoprotein M	Nucleus, nucleolus

	OS=Homo sapiens GN=HNRNPM PE=1 SV=3	
48.	Heterogeneous nuclear ribonucleoprotein R OS=Homo sapiens GN=HNRNPR PE=1 SV=1	Nucleus, microsome, nucleus, nucleoplasm, cytoplasm
49.	Heterogeneous nuclear ribonucleoprotein U-like protein 2 OS=Homo sapiens GN=HNRNPUL2 PE=1 SV=1	Nucleus
50.	Heterogeneous nuclear ribonucleoproteins A2/B1 OS=Homo sapiens GN=HNRNPA2B1 PE=1 SV=2	Nucleus, nucleoplasm, cytoplasmic granule, secreted, exosome
51.	Heterogeneous nuclear ribonucleoproteins C1/C2 OS=Homo sapiens GN=HNRNPC PE=1 SV=4	Nucleus
52.	Histone H2A type 1-D OS=Homo sapiens GN=HIST1H2AD PE=1 SV=2	Nucleus, chromosome
53.	Histone-binding protein RBBP4 OS=Homo sapiens GN=RBBP4 PE=1 SV=3	Nucleus
54.	Histone-binding protein RBBP7 OS=Homo sapiens GN=RBBP7 PE=1 SV=1	Nucleus
55.	Inhibitor of nuclear factor kappa-B kinase-interacting protein OS=Homo sapiens GN=IKBIP PE=1 SV=1	Endoplasmic reticulum membrane, single-pass membrane protein
56.	Interleukin enhancer-binding factor 2 OS=Homo sapiens GN=ILF2 PE=1 SV=2	Nucleus, nucleolus, cytoplasm, nucleus
57.	Interleukin enhancer-binding factor 3 OS=Homo sapiens GN=ILF3 PE=1 SV=3	Nucleus, nucleolus, cytoplasm, nucleus
58.	Lamin-B1 OS=Homo sapiens GN=LMNB1 PE=1 SV=2	Nucleus inner membrane, lipid-anchor, nucleoplasmic side
59.	Lamin-B2 OS=Homo sapiens GN=LMNB2 PE=1 SV=3	Nucleus inner membrane, lipid-anchor, nucleoplasmic side
60.	Lanosterol synthase OS=Homo sapiens GN=LSS PE=1 SV=1	Endoplasmic reticulum membrane, peripheral membrane protein
61.	Mitochondrial 10-formyltetrahydrofolate dehydrogenase OS=Homo sapiens GN=ALDH1L2 PE=1 SV=2	Mitochondrion
62.	Mitochondrial inner membrane protein OS=Homo sapiens GN=IMMT PE=1 SV=1	Mitochondrion inner membrane, multi-pass membrane protein
63.	NADH dehydrogenase [ubiquinone] iron-sulfur protein 2, mitochondrial OS=Homo sapiens GN=NDUFS2 PE=1 SV=2	Mitochondrion inner membrane, peripheral membrane protein, matrix side
64.	NADH dehydrogenase [ubiquinone] iron-sulfur protein 8, mitochondrial OS=Homo sapiens GN=NDUFS8 PE=1 SV=1	Mitochondrion
65.	NADPH--cytochrome P450 reductase OS=Homo sapiens GN=POR PE=1 SV=2	Endoplasmic reticulum membrane, single-pass membrane protein, cytoplasmic side

66.	Nesprin-3<br>OS=Homo sapiens GN=C14orf49 PE=1 SV=2	Nucleus outer membrane, single-pass type IV membrane protein, nucleus envelope, rough endoplasmic reticulum
67.	Neuroblast differentiation-associated protein AHNAK<br>OS=Homo sapiens GN=AHNAK PE=1 SV=2	Nucleus
68.	Nicastrin<br>OS=Homo sapiens GN=NCSTN PE=1 SV=2	Membrane, single-pass type I membrane protein, melanosome
69.	Niemann-Pick C1 protein<br>OS=Homo sapiens GN=NPC1 PE=1 SV=2	Late endosome membrane, multi-pass membrane protein, lysosome membrane, multi-pass membrane protein
70.	Nodal modulator 1<br>OS=Homo sapiens GN=NOMO1 PE=1 SV=5	Membrane, single-pass type I membrane protein
71.	Non-POU domain-containing octamer-binding protein<br>OS=Homo sapiens GN=NONO PE=1 SV=4	Nucleus, nucleus, nucleolus, nucleus speckle
72.	Nuclear mitotic apparatus protein 1<br>OS=Homo sapiens GN=NUMA1 PE=1 SV=2	Nucleus matrix, chromosome, cytoplasm, cytoskeleton, spindle pole, cytoplasm, cytoskeleton, microtubule organizing center, centrosome
73.	Nuclear pore glycoprotein p62<br>OS=Homo sapiens GN=NUP62 PE=1 SV=3	Nucleus, nuclear pore complex, cytoplasm, cytoskeleton, spindle pole
74.	Nucleolin<br>OS=Homo sapiens GN=NCL PE=1 SV=3	Nucleus, nucleolus, cytoplasm
75.	Nucleoporin Nup43<br>OS=Homo sapiens GN=NUP43 PE=1 SV=1	Chromosome, centromere, kinetochore, nucleus, nuclear pore complex
76.	Nucleoprotein TPR<br>OS=Homo sapiens GN=TPR PE=1 SV=3	Nucleus, nucleus membrane, peripheral membrane protein, nucleoplasmic side, nucleus envelope, nucleus, nuclear pore complex, cytoplasm, cytoplasm, cytoskeleton, spindle, chromosome, centromere, kinetochore, nucleus membrane, peripheral membrane protein, cytoplasmic side
77.	Nucleoside diphosphate kinase B<br>OS=Homo sapiens GN=NME2 PE=1 SV=1	Cytoplasm, nucleus, cell projection, lamellipodium, cell projection, ruffle
78.	Ornithine aminotransferase, mitochondrial<br>OS=Homo sapiens GN=OAT PE=1 SV=1	Mitochondrion matrix
79.	Polyadenylate-binding protein 3<br>OS=Homo sapiens GN=PABPC3 PE=1 SV=2	Cytoplasm
80.	Polyadenylate-binding protein 4<br>OS=Homo sapiens GN=PABPC4 PE=1 SV=1	Cytoplasm
81.	Probable ATP-dependent RNA helicase DDX5<br>OS=Homo sapiens GN=DDX5 PE=1 SV=1	Nucleus, nucleolus
82.	Procollagen-lysine,2-oxoglutarate 5-dioxygenase 1<br>OS=Homo sapiens GN=PLOD1 PE=1 SV=2	Rough endoplasmic reticulum membrane, peripheral membrane protein, lumenal side
83.	Procollagen-lysine,2-oxoglutarate 5-dioxygenase 2<br>OS=Homo sapiens GN=PLOD2 PE=1 SV=2	Rough endoplasmic reticulum membrane, peripheral membrane protein, lumenal side

84.	Procollagen-lysine,2-oxoglutarate 5-dioxygenase 3<br>OS=Homo sapiens GN=PLOD3 PE=1 SV=1	Rough endoplasmic reticulum membrane, peripheral membrane protein, lumenal side
85.	Prolyl 4-hydroxylase subunit alpha-2<br>OS=Homo sapiens GN=P4HA2 PE=1 SV=1	Endoplasmic reticulum lumen
86.	Protein ERGIC-53<br>OS=Homo sapiens GN=LMAN1 PE=1 SV=2	Endoplasmic reticulum-Golgi intermediate compartment membrane, single-pass type I membrane protein, Golgi apparatus membrane, single-pass membrane protein, endoplasmic reticulum membrane, single-pass type I membrane protein
87.	Protein NOXP20<br>OS=Homo sapiens GN=FAM114A1 PE=1 SV=2	Cytoplasm
88.	Protein transport protein Sec23A<br>OS=Homo sapiens GN=SEC23A PE=1 SV=2	Smooth endoplasmic reticulum membrane, peripheral membrane protein, Golgi apparatus membrane, peripheral membrane protein
89.	Pyruvate dehydrogenase E1 component subunit beta, mitochondrial<br>OS=Homo sapiens GN=PDHB PE=1 SV=3	Mitochondrion matrix
90.	Ras-related protein Rab-11A<br>OS=Homo sapiens GN=RAB11A PE=1 SV=3	Cell membrane, lipid-anchor, recycling endosome membrane, lipid-anchor, cleavage furrow, cytoplasmic vesicle, phagosome
91.	Ras-related protein Rab-2A<br>OS=Homo sapiens GN=RAB2A PE=1 SV=1	Endoplasmic reticulum-Golgi intermediate compartment membrane, lipid-anchor, melanosome, endoplasmic reticulum membrane, lipid-anchor, Golgi apparatus membrane, lipid-anchor
92.	Ras-related protein Rab-2B<br>OS=Homo sapiens GN=RAB2B PE=1 SV=1	Cell membrane, lipid-anchor, cytoplasmic side, endoplasmic reticulum membrane, Golgi apparatus membrane
93.	Ras-related protein Rab-6A<br>OS=Homo sapiens GN=RAB6A PE=1 SV=3	Golgi apparatus membrane, lipid-anchor<br>Isoform 1 : Golgi apparatus membrane, lipid-anchor<br>Isoform 2 : Golgi apparatus membrane, lipid-anchor
94.	Ribosome-binding protein 1<br>OS=Homo sapiens GN=RRBP1 PE=1 SV=4	Endoplasmic reticulum membrane, single-pass type III membrane protein
95.	RuvB-like 2<br>OS=Homo sapiens GN=RUVBL2 PE=1 SV=3	Nucleus matrix, nucleus, nucleoplasm, cytoplasm, membrane
96.	Sec1 family domain-containing protein 1<br>OS=Homo sapiens GN=SCFD1 PE=1 SV=4	Cytoplasm, endoplasmic reticulum membrane, peripheral membrane protein, Golgi apparatus, Golgi stack membrane, peripheral membrane protein
97.	Serum albumin<br>OS=Homo sapiens GN=ALB PE=1 SV=2	Secreted
98.	Signal transducer and activator of transcription 1-alpha/beta<br>OS=Homo sapiens GN=STAT1 PE=1 SV=2	Cytoplasm, nucleus
99.	Single-stranded DNA-binding protein, mitochondrial<br>OS=Homo sapiens GN=SSBP1 PE=1 SV=1	Mitochondrion, mitochondrion matrix, mitochondrion nucleoid

100.	Splicing factor 3B subunit 3<br>OS=Homo sapiens GN=SF3B3 PE=1 SV=4	Nucleus
101.	Splicing factor, proline- and glutamine-rich<br>OS=Homo sapiens GN=SFPQ PE=1 SV=2	Nucleus matrix, cytoplasm
102.	Staphylococcal nuclease domain-containing protein 1<br>OS=Homo sapiens GN=SND1 PE=1 SV=1	Cytoplasm, nucleus, melanosome
103.	Stomatin-like protein 2<br>OS=Homo sapiens GN=STOML2 PE=1 SV=1	Cell membrane, peripheral membrane protein, mitochondrion, mitochondrion inner membrane, lipid-anchor, mitochondrion intermembrane space, membrane raft, cytoplasm, cytoskeleton
104.	Succinate dehydrogenase [ubiquinone] flavoprotein subunit, mitochondrial<br>OS=Homo sapiens GN=SDHA PE=1 SV=2	Mitochondrion inner membrane, peripheral membrane protein, matrix side
105.	Succinate dehydrogenase [ubiquinone] iron-sulfur subunit, mitochondrial<br>OS=Homo sapiens GN=SDHB PE=1 SV=3	Mitochondrion inner membrane, peripheral membrane protein, matrix side
106.	Succinyl-CoA ligase [GDP-forming] subunit beta, mitochondrial<br>OS=Homo sapiens GN=SUCLG2 PE=1 SV=2	Mitochondrion
107.	T-complex protein 1 subunit zeta-2<br>OS=Homo sapiens GN=CCT6B PE=1 SV=5	Cytoplasm
108.	Thioredoxin domain-containing protein 5<br>OS=Homo sapiens GN=TXNDC5 PE=1 SV=2	Endoplasmic reticulum lumen
109.	Thioredoxin-related transmembrane protein 1<br>OS=Homo sapiens GN=TMX1 PE=1 SV=1	Membrane, single-pass type I membrane protein, endoplasmic reticulum membrane, single-pass type I membrane protein
110.	Torsin-1A-interacting protein 1<br>OS=Homo sapiens GN=TOR1AIP1 PE=1 SV=2	Nucleus inner membrane, single-pass membrane protein
111.	Transmembrane emp24 domain-containing protein 4<br>OS=Homo sapiens GN=TMED4 PE=1 SV=1	Endoplasmic reticulum membrane, single-pass type I membrane protein
112.	Ubiquitin-conjugating enzyme E2 variant 1<br>OS=Homo sapiens GN=UBE2V1 PE=1 SV=2	Nucleus
113.	UDP-glucose:glycoprotein glucosyltransferase 1<br>OS=Homo sapiens GN=UGGT1 PE=1 SV=3	Endoplasmic reticulum lumen, endoplasmic reticulum-Golgi intermediate compartment
114.	UPF0568 protein C14orf166<br>OS=Homo sapiens GN=C14orf166 PE=1 SV=1	Nucleus, cytoplasm, cytosol, cytoplasm, perinuclear region, cytoplasm, cytoskeleton, microtubule organizing center, centrosome
115.	UTP--glucose-1-phosphate uridylyltransferase<br>OS=Homo sapiens GN=UGP2 PE=1 SV=5	Cytoplasm
116.	Vacuolar protein sorting-associated protein 35<br>OS=Homo sapiens GN=VPS35 PE=1 SV=2	Cytoplasm, membrane, peripheral membrane protein, endosome, early endosome, late endosome

117.	Vesicle-trafficking protein SEC22b<br>OS=Homo sapiens GN=SEC22B PE=1 SV=4	Endoplasmic reticulum membrane, single-pass type IV membrane protein, endoplasmic reticulum-Golgi intermediate compartment membrane, Golgi apparatus, cis-Golgi network membrane, Golgi apparatus, trans-Golgi network membrane, melanosome
118.	Vesicular integral-membrane protein VIP36<br>OS=Homo sapiens GN=LMAN2 PE=1 SV=1	Endoplasmic reticulum-Golgi intermediate compartment membrane, single-pass type I membrane protein, Golgi apparatus membrane, single-pass membrane protein, endoplasmic reticulum membrane, single-pass type I membrane protein
119.	Voltage-dependent anion-selective channel protein 2<br>OS=Homo sapiens GN=VDAC2 PE=1 SV=2	Mitochondrion outer membrane
120.	X-ray repair cross-complementing protein 5<br>OS=Homo sapiens GN=XRCC5 PE=1 SV=3	Nucleus, nucleus, nucleolus, chromosome
121.	X-ray repair cross-complementing protein 6<br>OS=Homo sapiens GN=XRCC6 PE=1 SV=2	Nucleus, chromosome
